# Effect of Combination Therapy of Methotrexate with Vitamin A in Patients with Low Risk GTN (Gestational Trophoblastic Neoplasia)

**Published:** 2018

**Authors:** Sedigheh Ghasemian, Zohreh Yousefi, Marjaneh Farazestanian, Leila Mousavi Seresht, Mohsen Foroughipour, Saeid Akhlaghi

**Affiliations:** a *Department of Obstetrics and Gynecology, School of Medicine, Urmia University of Medical Sciences, Urmia, Iran.*; b *Department of Obstetrics and Gynecology, Fellowship of Gynecologist Oncologist, School of Medicine, Mashhad University of Medical Sciences, Mashhad, Iran. *; c *Department of Obstetrics and Gynecology, School of Medicine, Mashhad University of Medical Sciences, Mashhad, Iran. *; d *School of Medicine, Mashhad University of Medical Sciences, Mashhad, Iran. *; e *School of Medicine, Mashhad University of Medical Sciences, Mashhad, Iran. *; f *School of Medicine, Mashhad University of Medical Sciences, Mashhad, Iran.*

**Keywords:** Methotrexate, Vitamin A, Gestational Trophoblastic neoplasia (GTN), B-HCG, Combination therapy.

## Abstract

Methotrexate as a single agent chemotherapy in most women with low risk gestational trophoblastic neoplasia (GTN) has been associated with high treatment rate. Combination of methotrexate with Vitamin A due to reduced number of chemotherapy regime courses is one of the treatment options for patients with low-risk GTN**. ** Therefore, this study was performed with aim to determine the efficacy of combination therapy of Methotrexate with Vitamin A in low risk GTN treatment. This randomized clinical trial was performed on 49 patients with low risk gestational trophoblastic neoplasia. The treatment group (Group A = 19 cases) weekly received Methotrexate 50 mg/m^2^, and Vitamin A 200000 IU, intra-muscular, and the control group (Group B = 30 cases) only received Methotrexate 50 mg/m^2^ weekly. All patients were followed up for 8 weeks. Then, treatment outcomes were compared between two groups, and response to therapy was assessed in two groups by evaluation of HCG serum level. P < 0.05 was considered significant.Mean of B-HCG serum level after 4 weeks in Group A and Group B was 68.5 mIu/mL and 360 mIu/mL, respectively (*P* = 0.018), and after 8 weeks was 1 mIu/mL and 12 mIu/mL, respectively (*P* = 0.074). Combination therapy of Methotrexate and Vitamin A in low risk GTN is associated with shorter duration of chemotherapy.

## Introduction

Low risk gestational trophoblastic neoplasia (GTN) is a type of malignant tumor which has 80% remission with primary single chemotherapy that requires several courses of chemotherapy and long-terms follow-up with monitoring of B-HCG level (1). GTN, irrespective of the site and gestational age, may develop after a molar or non-molar pregnancy as a consequence of autonomous overgrowth of one of the three cell layers of the trophoblast (2). To achieve complete remission (B-HCG level < 5 mIu/mL), the time required is usually 4-6 months, which is time-consuming and no cost benefit (3). Metastatic GTN occurs in 4% of patients with complete hydatidiform mole after the evacuation of molar tissue. Cell proliferation and differentiation is controlled by genes on cell cycle, and abnormalities in this cycle are repaired by some genes, when repairing failed, apoptosis (programmed cell death) occurs. So, failure in apoptosis leads to growth of neoplastic cell. In human, P53 gene is located on the short arm of chromosome 17 (17p13.1). Many cancer cells inactivate P53 gene, allowing the cells evade death and continue proliferating up to becoming a tumor (4). Vitamin A has an important role in regulation of cell proliferation, differentiation and apoptosis by increasing the activity of P53 that caused G1 phase arrest and Bcl-2 gens which encourages apoptosis (5). The result of apoptosis is a protective mechanism for malignancy. In patients with low levels of Vitamin A, disruption of controlling cell proliferation and differentiation occurs (6, 7). Administration of Vitamin A in trophoblastic neoplasia may increase the process of apoptosis. 

**Table 1:** Characteristics of patients in Group A and Group B.

**Characteristics**	**Group B (N = 29)**	**Group A (N = 14)**	**P-value**
Patients age	25.86 ± 5.95	25.92 ± 6.34	0.973
Gestational age	9.48 ± 2.62	10.57 ± 2.06	0.182
Previous mole	3.4 %	7.2 %	1
Incomplete mole Complete mole	10 (34.5) % 19 (65.5 %)	7 (50 % ) 7 (50 %)	0.507
Pre-Therapy B-HCG	7270 mIU/mL	4580 mIU/mL	0.551
Stage 1	18 (62.1 %)	12 (85.7 %)	0.164
Stage 3	11 (37.9 %)	2 (14.3 %)	

**Table 2:** Side effects of Methotrexate & Vitamin A

**Side Effects**	**Group A**	**Group B**
Headache & Visual Loss	1	o
Skin Lesion	0	0
Liver Function Test Disorder	1	1	

**Table 3:** Complete remission and occurrence of Methotrexate resistance

**Groups**	**Complete Remission**	**Methotrexate Resistance**
Group A	8 (47.05 %)	3 (17.64 %)
Group B	12 (41.36 %)	0

**Figure 1:**
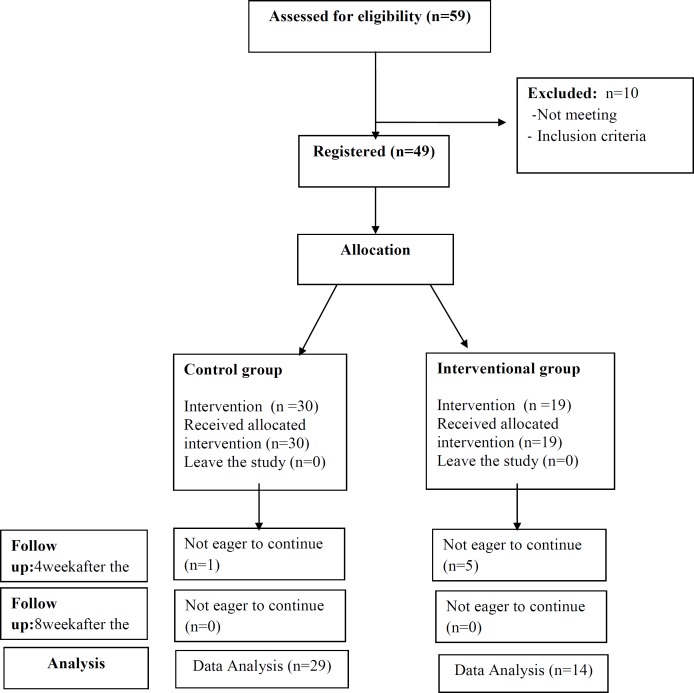
CONSORT flow chart of participants

Andrijono *et al.* in a study performed in 2007 reported that low retinol level in the liver and blood of patients with hydatidiform mole resulted in decreasing the retinoic acid in cell leading to uncontrolled proliferation of trophoblastic cells and reduction in apoptosis, and consequently increasing the risk of transformation of hydatidiform mole to GTN. They demonstrated that high dose of Vitamin A could be used for preventing of malignant transformation (8). 

In addition, a recent randomized controlled trial (RCT) has shown that Vitamin A prophylaxis may reduce the risk of malignant transformation (9). In clinical study of Sutanto *et al.,* combination therapy of per-oral Methotrexate with Vitamin A was effective in low risk GTN treatment (10). Since various treatment regimens are used for low-risk GTN treatment, yet the comparative benefits and risks of these regimens is unclear, therefore, this study was performed with the aim of evaluating the efficacy of combination therapy of Methotrexate and Vitamin A in low risk GTN treatment. 

## Experimental

*Materials and Methods*

This randomized clinical trial study was performed on 49 patients with low risk GTN referred to

 oncology department of Ghaem Hospital, Mashhad University of Medical Sciences in 2013-2014. The patients with rise or Plateau of serum β-HCG level who were candidate for chemotherapy were selected. Inclusion criteria included: disease stage 1-3, score < 7, and no medical disease (skin lesion, hematologic, cerebral, liver, pulmonary, and renal disease). The variables included: age, gestational age, parity, type of mole, previous mole, GTN stage, pre-therapy levels of β-HCG, and weekly serial levels of β-HCG. The patients were divided into two groups: treatment group (Group A = 19 cases) that received weekly intra-muscular Methotrexate 50 mg/m^2^ with Vitamin A 200000 IU, and control group (Group B) that only received weekly Methotrexate 50 mg/m^2^. Evaluation of B-HCG serum level was performed with radioimmuno assay (RIA). The treatment was continued for two consequent weeks until B-HCG serum level became normal value. Patients with Methotrexate resistance and the cases who showed side-effects of Vitamin A and Methotrexate including SGOT and SGPT increased value, skin lesion, visual loss, headache, and vomiting were excluded from the study. Finally, 29 patients in Group A and 14 in Group B were analyzed (Figure 1). The response to therapy in two groups was weekly assessed by evaluation of HCG serum level. This intervention was performed for eight weeks. The Pre- treatment mean value of β-HCG level and weekly serial B-HCG level were assessed up to eight weeks in two groups, and finally, β-HCG level of 4th and 8th week were compared with pre-treatment β-HCG. Data was analyzed by SPSS software (version 18), and Man-Whitney and t-test. *P* < 0.05 was considered significant.

## Results

Characteristics of patients are described in Table 1. none of these variables were significantly different in two groups. The response to therapy in two groups was assessed by titer of β-HCG serum level. Serum β-HCG level was assessed pre-treatment, 4 weeks, and 8 weeks after treatment (Table 2). The obtained results showed that mean Pre- treatment level of β-HCG was 4580 mIU/mL in Group A and 7270 mIU/mL in Group B (P = 0.551) . Mean β-HCG level 4 weeks after treatment was 68.5 mIU/mL in Group A and 360 mIU/mL in Group B (P = 0.018). Mean β-HCG level 8 weeks after treatment was 1 mIU/mL in Group A and 12 mIU/mL (P = 0.074) in Group B. Analysis with Man-Whitney Test showed that β-HCG level 4 weeks after treatment was significantly lower in Group A than Group B (P = 0.018). But, 8 weeks after treatment, the difference of β-HCG level in two groups was near to significant (P = 0.074). Administration of high dose of vitamin A (200,000 IU) may lead to increasing Vitamin A related side effects. Evaluation of side effects in Group A showed one patient with headache and visual loss with confirm of papilla edema consequently by Vitamin A, and one patient with increased levels of SGOT and SGPT. In Group B, only one case of elevated SGOT and SGPT was observed. These three cases were excluded from the study (Table 2). Furthermore, assessment of response to therapy showed that 8 patients (47.05%) in Group A and 12 (41.36%) 

in Group B achieved complete remission. In treatment group, three patients had resistance to therapy who excluded from this study**. (**Table 3). 

## Discussion

The great interest relationship between Vitamin A and hydatidiform mole was reported in the epidemiological studies (9). In attention to the role of Vitamin A in prevention of post-mole malignant trophoblastic disease and regarding to adjuvant therapy of GTN in previous studies, despite of high dose daily administration of Vitamin A in some studies (8, 10), in our study, dose of Vitamin A was weekly 200000 IU and lower than their dose. In our clinical trial, complete remission was observed in 47.05% after only eight doses of Methotrexate and Vitamin A, but in the study of Sutanto *et al.,* 20 % complete remission was obtained after twelve doses of Methotrexate and daily Vitamin A (100000 IU) for three cycles (10). The better and earlier complete remission in our study may be related to by intra-muscular administration of Vitamin A and its effective absorption. Combination of vitamin A 100,000 IU and methotrexate in the study of Sutanto led to accelerated β-hCG serum declination in treatment of patients with low risk GTN (10). Although the results of Andrijonos showed that the rate of malignant trophoblastic disease (MTD) was reduced in the group receiving vitamin A therapy. On the other hand, in the study of Sutanto, mean level of pre-treatment β-HCG in treatment group was 59349.5 mIU/mL, but in our study, it was 4580 mIU/mL, this significant difference may be due to different pre-treatment β-HCG level in two studies.

In our clinical trial, one patient had papilla edema due to Vitamin A, but this side effect wasn`t reported in Sutanto›s study. It can be told that this side effect is resulted from more absorption of Vitamin A by intra-muscular administration. Various studies have been performed to evaluate the efficacy of weekly administration of IM MTX (11, 12). Finally, combination of Vitamin A and Methotrexate in low risk GTN treatment accelerates the response to therapy with decreased β-HCG serum levels 4 weeks after the intervention. Some patients with hydatidiform mole suffer from long-term Vitamin A deficiency, so serum level of Vitamin A or retinol should be evaluated before combination-therapy (13). However, multicenter collaboration studies are necessary to confirm the effective dose of Vitamin A. 

## Conclusion

Vitamin A increases regression of trophoblastic cells and decreases β-HCG level. Also, Vitamin A increases the efficacy of methotrexate inducing more malignant cell death than using Methotrexate alone.
